# Dissection extending from extra- to intracranial arteries: A case report of progressive ischemic stroke

**DOI:** 10.1097/MD.0000000000006980

**Published:** 2017-05-26

**Authors:** Fu-Liang Zhang, Zhen-Ni Guo, Yang Liu, Yun Luo, Yi Yang

**Affiliations:** a Stroke Center; b Neuroscience Center, Department of Neurology; c Department of Radiology, the First Hospital of Jilin University, Chang Chun, China.

**Keywords:** case report, cervical artery dissections, 3D fat-saturated T1 VISTA imaging, intracranial artery dissections

## Abstract

**Rationale:**

Cervicocephalic artery dissections, once considered a rare disease, have become increasingly recognized as a cause of stroke in young and middle-aged individuals. Early diagnosis is mandatory because anticoagulation or antithrombotic therapy can help prevent primary or secondary ischemic events. However, the diagnosis is still a crucial challenge for radiologists and neurologists.

**Patient concerns:**

We reported a rare case of 33-year-old patient with progressive ischemic stroke due to dissection from an intimal tear in the right proximal internal carotid artery to distal middle cerebral artery.

**Diagnoses:**

3D fat-saturated T1 VISTA imaging, owing to its comprehensive neck and head coverage, high spatial resolution, enables the reader to have several sections with good contrast covering the dissected arterial segment, even in the rare dissection involving extra- and intra-cranial arteries referred in this article.

**Interventions:**

Clopidogrel 75mg daily was prescribed, also the patient was given rehabilitation training.

**Outcomes:**

His symptoms improved gradually.

**Lessons:**

We describe that 3D fat-saturated T1 VISTA was helpful for the diagnosis and follow-up in our case of cervicocephalic artery dissection complicated with progressive ischemic stroke. However, for totally acute occlusion of the artery without typical features of dissection, the unequivocal distinction between intramural haematoma and intraluminal thrombus may still be difficult with 3D fat-saturated T1 VISTA alone. Future studies should investigate whether an optimal VISTA technique would be useful for making a definite diagnosis.

## 1 Introduction

Cervical artery dissection (CAD) and intracranial artery dissection (IAD) are always diagnostic challenges for radiologists and neurologists. They are among the most common causes of stroke in young and middle-aged adults.^[1–3]^ We present a rare case that illustrates the value of 3D fat-saturated T1 volumetric isotropic turbo spin echo acquisition (VISTA) imaging with fat suppression to explore the entirety of extra- and intracranial arteries on a 3.0 T MRI.

## 2 Case report

**Figure 1:**
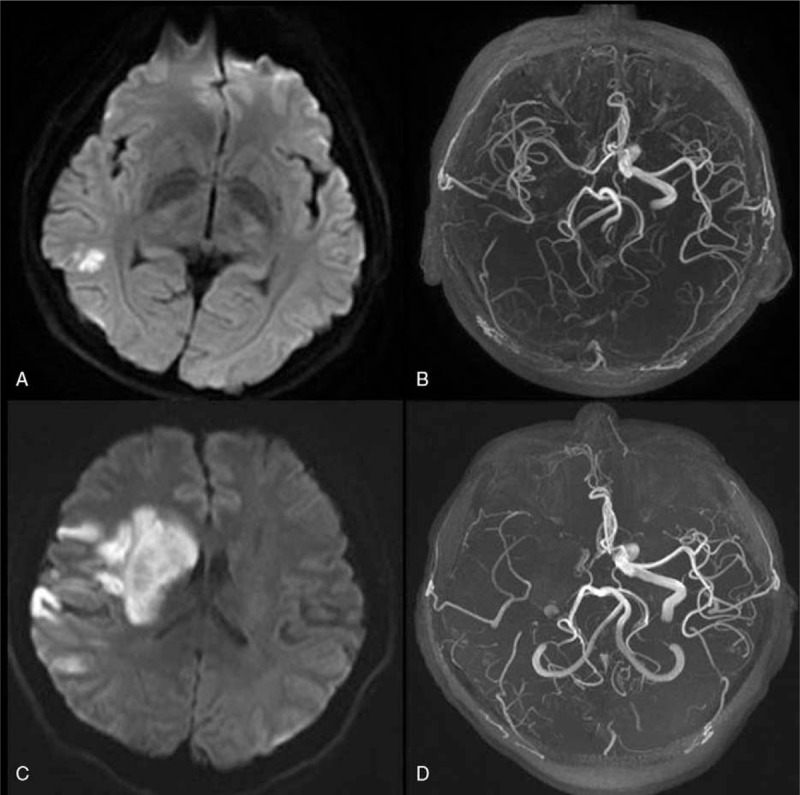
Imaging examination of the brain and cerebral vessels. DWI (A) showed increased signal intensity, indicating acute infarction in the right postwatershed area. MRA (B) demonstrated severe stenosis versus occlusion of the right internal carotid artery. Repeat DWI and MRA (C, D) revealed additional infarction in the right basal ganglia and the occlusion of the right middle cerebral artery. DWI = diffusion weighted imaging, MRA = magnetic resonance angiography.

**Figure 2:**
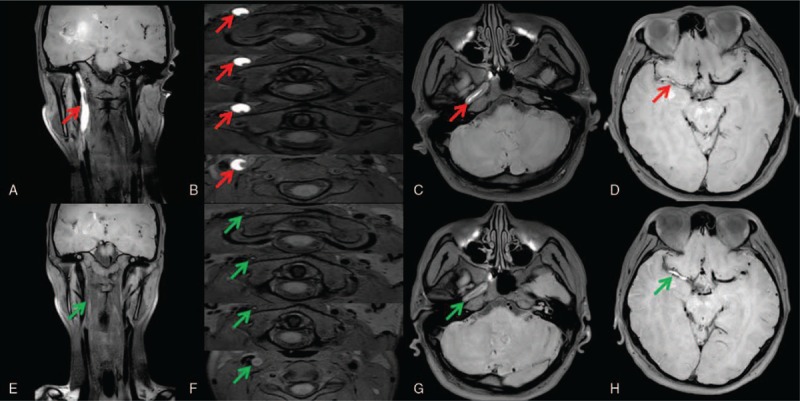
3D fat-saturated T1 VISTA imaging of the dissection before and after therapy. 3D fat-saturated T1 VISTA imaging (A–D red arrow) demonstrated a continuous crescentic high signal of the right internal carotid artery, and elongated, round high signal of the right middle cerebral artery, indicating the probable diagnosis of subacute dissection extending from the origin of RICA to distal RMCA. A repeat imaging (E–H green arrow) 7 months later revealed that the signal intensity of right internal carotid artery decreased, while the signal of right middle cerebral artery became higher than before, indicating persistent occlusion of the artery. RICA = right internal carotid artery, RMCA = right middle cerebral artery, VISTA = volumetric isotropic turbo spin echo acquisition.

A 33-year-old man presented to the neurology department with right-side headache and left-side hemiparesis. On examination, no murmurs were appreciated on cardiac auscultation, and pulses were symmetric. Neurological examination demonstrated a central left-sided hemiparesis (the upper limb: MRC grade 3+/5, the lower limb: MRC grade 4/5). Brain diffusion-weighted imaging (Fig. 1A) at admission showed acute ischemic infarction in the right temporal lobe, and magnetic resonance angiography (MRA) (Fig. 1B) demonstrated severe stenosis versus occlusion of the right internal carotid artery (RICA), which territory was supplied by the anterior and the right posterior communicating artery. He did not have conventional stroke risk factors such as hypertension, diabetes, and hyperlipidemia or a history of cardiac valvar disease, atrial fibrillation, or features of inherited connective tissue disorder. His symptoms were worsening 2 days later; however, repeat diffusion-weighted imaging and MRA (Fig. 1C and D) revealed additional infarction in the right basal ganglia and the occlusion of the right middle cerebral artery (RMCA). Further investigation was performed to identify the cause of the occlusion. 3D fat-saturated T1 VISTA imaging (Fig. 2A–D) demonstrated a continuous crescentic high signal of the RICA, and elongated, round high signal of the RMCA, indicating the probable diagnosis of subacute dissection extending from the origin of RICA to distal RMCA. Clopidogrel 75 mg daily was prescribed, also he was given rehabilitation training, and his symptoms improved gradually. A follow-up imaging examination (Fig. 2E–H) 7 months later revealed that the intramural hematoma signal intensity of RICA decreased notably, while that of RMCA became brighter noticeably than before, suggesting that the speed of intramural hematoma decomposition might differed at different segments of the artery.

## 3 Discussion

CADs and IADs are among the most common causes of stroke in young and middle-aged adults without vascular risk factors who are less likely to have cerebrovascular atherosclerosis. CADs accounted for 8% to 25% of stroke in patients under 45 years, and approximately 2% of ischemic strokes overall,^[1,4]^ while IADs were estimated to represent <10% of all spontaneous cervicocephalic artery dissections in the adults.^[3]^

Trauma, infections, and connective tissue disorders are well-known predisposing factors, but the majority of cases are spontaneous or related to trauma, in a healthy artery, with no identifiable etiological factor.^[1,3,5,6]^ The patient referred in the text had no history of trauma, but reported coughing and sneezing caused by cold previously.

Most common type of CADs have been reported to occur in the internal carotid artery, more than 2 cm after the bifurcation in the majority, and may extend in height to the sub- and intrapetrous segments, but rarely as far as the intracranial segments,^[6]^ as the rare case mentioned in the text manifested.

Early and reliable diagnosis is of great importance to start a timely appropriate management to prevent primary or secondary ischemic events.^[2,6]^ The typical patient with CADs presents with pain on one side of head or neck accompanied by partial Horner syndrome and followed by cerebral ischemia hours or days later. This classic triad is found in less than one third of patients, but the presence of any 2 elements of this triad should strongly suggest the diagnosis.^[1,4]^ Apart from right-side headache, the patient described manifested as progressive ischemic stroke for the extension of dissection from an intimal tear in the proximal RICA to distal middle cerebral artery, which resulted in severe arterial stenosis and secondary ischemia.

Digital subtraction angiography is traditionally considered the golden standard for dissection diagnosis due to the precise delineation of the luminal abnormalities, but its use is limited by its invasiveness and cost. This technique does not enable the detection of the mural hematoma either, angiography may be considered normal in the event of CAD and IAD, especially if the dissection does not cause any alteration in the arterial lumen.^[4,6]^

Recent magnetic resonance technologic innovations allow the acquisition of 3D fat-saturated T1 VISTA imaging,^[7,8]^ using variable refocusing flip-angle turbo-spin-echo imaging, with large and complete coverage of the head and neck area and acceptable scan duration for clinical practice. The parameters of the imaging sequences were as follows: an oblique coronal plane acquisition, spectral adiabatic inversion recovery fat saturation mode, repetition time (TR)/echo time (TE) = 350/19 ms, field of view = 280 × 199 × 120 cm^3^, 400 × 284 matrix, variable refocusing flip angle, slice interval = 0; voxel size = 0.7 × 0.7 × 0.7 cm^3^, oversample factor = 1.5, and number of excitations = 2. The contrast between the hyper-T1 signal of the hematoma and the surrounding tissues, associated with a high spatial resolution, enables the reader to have several sections with good contrast covering the dissected arterial segment, even in the rare dissection extending from extra- to intracranial arteries referred in the text. Many studies have investigated the value of 3D T1 VISTA for the diagnosis of cervicocephalic artery dissections, and all of them showed that 3D T1 VISTA is more useful than conventional imaging tools. Takemoto et al^[7]^ first reported the value of 3D T1 VISTA sequence in the diagnosis of CAD. They concluded that a 3D black blood T1-weighted imaging yielded better distinction of intramural hematoma in intracranial vertebral artery dissection compared to 2D spin echo T1-weighted images and time-of-flight MRA.^[9]^ Another study^[8]^ showed that 3D T1 VISTA is useful for diagnosing vertebrobasilar artery dissections at subacute stages, as it can reveal vessel wall and lumen abnormalities, including not only intramural hematomas, but also intimal flaps, dilatation, and abnormal vessel enhancement, with no or minimum flow artifacts due to sequence-endogenous flow void enhancement. Also 3D fat-saturated T1 VISTA imaging can be used as follow-up imaging examination according to the signal intensity changes. The intramural hematoma showed a wide variety of signal intensities from mild to moderate and very bright hyperintensities, depending on the age of the hematoma; however, it differed from that of cerebral hematomas. These differences in the speed of hematoma decomposition might be explained by recurrent microbleeds into the intramural hematomas which are caused by leaky neovessels. As the follow-up imaging examination 7 months later of the patient referred in the text revealed, the intramural hematoma signal intensity of RICA decreased notably, while that of RMCA became brighter than before, suggesting that the speed of intramural hematoma decomposition might differed at different segments of the artery.

Although there were many advantages mentioned above for 3D T1 VISTA in the diagnosis of CAD, we also found the limitations of 3D T1 VISTA in the clinical practice. First, for totally acute occlusion of the artery without typical imaging features of dissection, the unequivocal distinction between intramural hematoma and intraluminal thrombus may still be difficult with 3D T1 VISTA alone. Just like the patient mentioned in this article, comprehensively considering the successional lesions starting from the proximal RICA to RMCA and typical crescent-like hyperintensity of the RICA, we deduced the probable diagnosis of subacute dissection extending from the origin of RICA to distal RMCA. However, we could not completely rule out the possibility of thrombus formation adjacent to the dissected RICA resulting in the occlusion of the RMCA. Second, spin-echo metrics are time consuming and this may be paramount in critical care scenarios, echo-planar imaging would offer faster acquisition in this condition.

It is reported that recanalization rate of CADs varies among studies.^[7,10]^ From 40% to 80%, and usually happens within the first 2 to 3 months, while others may leave sequelae on the vascular wall and arterial lumen, with varying degrees of residual stenosis, or even occlusion, as the follow-up 3D fat-saturated T1 VISTA imaging showed in the text.^[6,11]^

There were also several limitations in our study. First, this was a case report and the contrast-enhanced imaging was not performed. Contrast-enhanced 3D T1 VISTA might be useful in manifesting subtle structure abnormalities, assessing vessel inflammatory reaction, and distinguishing intramural hematoma from intraluminal thrombus. Second, as previous studies already showed that a 3D T1 VISTA can provide similar information and may be a substitute for 2D sequence, the 2D T1 black blood sequence was not performed for the patient.

In conclusion, we describe that 3D T1-VISTA was helpful for the diagnosis and follow-up in our case of cervicocephalic artery dissection complicated with progressive ischemic stroke. However, for totally acute occlusion of the artery without typical features of dissection, the unequivocal distinction between intramural hematoma and intraluminal thrombus may still be difficult with 3D T1 VISTA alone. Future studies should investigate whether an optimal VISTA technique would be useful for making a definite diagnosis.

## References

[1] Schievink WI Spontaneous dissection of the carotid and vertebral arteries. N Engl J Med 2001;344:898–906. 11259724 10.1056/NEJM200103223441206

[2] Baumgartner RW Arnold M Baumgartner I Carotid dissection with and without ischemic events: local symptoms and cerebral artery findings. Neurology 2001;57:827–32. 11552012 10.1212/wnl.57.5.827

[3] Guillon B Levy C Bousser MG Internal carotid artery dissection: an update. J Neurol Sci 1998;153:146–58. 9511874 10.1016/s0022-510x(97)00287-6

[4] Schwartz NE Vertinsky AT Hirsch KG Clinical and radiographic natural history of cervical artery dissections. J Stroke Cerebrovasc Dis 2009;18:416–23. 19900642 10.1016/j.jstrokecerebrovasdis.2008.11.016

[5] Rubinstein SM Peerdeman SM van Tulder MW A systematic review of the risk factors for cervical artery dissection. Stroke 2005;36:1575–80. 15933263 10.1161/01.STR.0000169919.73219.30

[6] Debette S Leys D Cervical-artery dissections: predisposing factors, diagnosis, and outcome. Lancet Neurol 2009;8:668–78. 19539238 10.1016/S1474-4422(09)70084-5

[7] Takemoto K Takano K Abe H The new MRI modalities “BPAS and VISTA” for the diagnosis of VA dissection. Acta Neurochir Suppl 2011;112:59–65. 21691989 10.1007/978-3-7091-0661-7_11

[8] Sakurai K Miura T Sagisaka T Evaluation of luminal and vessel wall abnormalities in subacute and other stages of intracranial vertebrobasilar artery dissections using the volume isotropic turbo-spin-echo acquisition (VISTA) sequence: a preliminary study. J Neuroradiol J Neuroradiol 2013;40:19–28. 22633047 10.1016/j.neurad.2012.02.005

[9] Takano K Yamashita S Takemoto K MRI of intracranial vertebral artery dissection: evaluation of intramural haematoma using a black blood, variable-flip-angle 3D turbo spin-echo sequence. Neuroradiology 2013;55:845–51. 23619699 10.1007/s00234-013-1183-4

[10] Habs M Pfefferkorn T Cyran CC Age determination of vessel wall hematoma in spontaneous cervical artery dissection: a multi-sequence 3T cardiovascular magnetic resonance study. J Cardiovasc Magn Reson 2011;13:76. 22122756 10.1186/1532-429X-13-76 PMC3283525

[11] Baracchini C Tonello S Meneghetti G Neurosonographic monitoring of 105 spontaneous cervical artery dissections: a prospective study. Neurology 2010;75:1864–70. 20962286 10.1212/WNL.0b013e3181feae5e

